# The Relationship Between Leadership Styles, Job Satisfaction, and Tenure Among Nursing Home Administrators

**DOI:** 10.1093/geroni/igab046.1445

**Published:** 2021-12-17

**Authors:** Carey Peerman

**Affiliations:** Radford University, Elliston, Virginia, United States

## Abstract

Long-term care is considered a subset of health care administration as the characteristics and leadership skills needed differ from other areas of health care. Leadership style directly relates to organizational development, success, and effectiveness. For study purposes, specific focus was placed upon determining the degree to which nursing home administrators (NHAs) perceived styles of leadership determined job satisfaction with tenure as an NHA. Perceptions of leadership style and levels of job satisfaction were determined using a non-experimental, quantitative design, specifically employing a survey research approach. The research instrument in this study, the MLQ, provided the data essential to addressing the research questions and accompanying hypotheses. The effect of study participant response to items on the MLQ associated with the research questions was assessed using univariate analysis of descriptive factors and inferential statistical techniques for statistical significance testing purposes. A total of 87% of study participants indicated that they perceived their leadership style as Nursing Home Administrators (NHA’s) was reflective of Transformational Leadership. However, findings indicated that participants who had a Transactional Leadership style were more likely than other types of leadership styles to select an NHA as a career path if given the opportunity to choose this line of work in the future.

